# Synthesis and Exfoliation of Calcium Organophosphonates for Tailoring Rheological Properties of Sodium Alginate Solutions: A Path toward Polysaccharide-Based Bioink

**DOI:** 10.1021/acs.biomac.3c00081

**Published:** 2023-05-30

**Authors:** Kateřina Kopecká, Lenka Vítková, Zuzana Kroneková, Lenka Musilová, Petr Smolka, Filip Mikulka, Klára Melánová, Petr Knotek, Martin Humeník, Antonín Minařík, Aleš Mráček

**Affiliations:** † SYNPO, a.s., S. K. Neumanna 1316, 532 07 Pardubice, Czech Republic; ‡ Department of General and Inorganic Chemistry, Faculty of Chemical Technology, University of Pardubice, Studentska 573, 53210 Pardubice, Czech Republic; § Joint Laboratory of Solid State Chemistry, Faculty of Chemical Technology, University of Pardubice, Studentska 84, 53210 Pardubice, Czech Republic; ∥ Department of Physics and Materials Engineering, Faculty of Technology, Tomas Bata University in Zlin, Vavreckova 5669, 76001 Zlin, Czech Republic; ⊥ Polymer Institute of Slovak academy of Sciences, Dubravska cesta 9, 84541 Bratislava, Slovak Republic; # National Institute of Rheumatic Diseases, Nabrezie I. Krasku 4, 921 12 Piestany, Slovak Republic; % Centre of Polymer Systems, Tomas Bata University in Zlin, tr. Tomase Bati 5678, 76001 Zlin, Czech Republic; & Joint Laboratory of Solid State Chemistry, Faculty of Chemical Technology, University of Pardubice, Studentska 84, 53210 Pardubice, Czech Republic; $ Lehrstuhl Biomaterialien, Universitat Bayreuth, Prof.-Rudiger-Bormann Strasse 1, 95447 Bayreuth, Germany

## Abstract

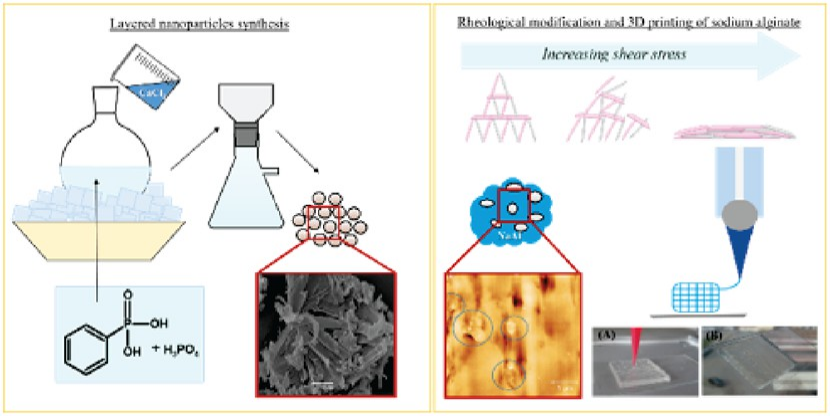

Layered nanoparticles with surface charge are explored as rheological modifiers for extrudable materials, utilizing their ability to induce electrostatic repulsion and create a house-of-cards structure. These nanoparticles provide mechanical support to the polymer matrix, resulting in increased viscosity and storage modulus. Moreover, their advantageous aspect ratio allows for shear-induced orientation and decreased viscosity during flow. In this work, we present a synthesis and liquid-based exfoliation procedure of phenylphosphonate–phosphate particles with enhanced ability to be intercalated by hydrophilic polymers. These layered nanoparticles are then tested as rheological modifiers of sodium alginate. The effective rheological modification is proved as the viscosity increases from 10^1^ up to 10^3^ Pa·s in steady state. Also, shear-thinning behavior is observed. The resulting nanocomposite hydrogels show potential as an extrudable bioink for 3D printing in tissue engineering and other biomedical applications, with good shape fidelity, nontoxicity, and satisfactory cell viability confirmed through encapsulation and printing of mouse fibroblasts.

## Introduction

3D printing has drawn a lot of attention due to the potential for precisely fabricating complex structures with minimal waste. Biocompatible materials are suggested for applications in the pharmaceutical and biomedical field, e.g., as drug delivery systems, tissue analogues for specialized *in vitro* testing, wound healing, and tissue engineering.^1^ Cell cultivation and tissue engineering, in particular, require a porous matrix with high water content in order to achieve close resemblance to the extracellular matrix and, thus, sustain cell functionality.^2^ To this end, natural polymer-based hydrogels—i.e., weakly cross-linked polymer networks capable of retaining large amounts of water^3^—appear as promising candidates in many of the suggested applications.

There have been recent developments in the processing of natural polymer-based hydrogels, which would allow additive manufacturing by means of extrusion 3D printing.^4,5^ The most established polymers include collagen,^6,7^ gelatin,^8−12^ hyaluronan,^12−14^ and sodium alginate (NaAlg).^8,10,14−16^ The wide usage of NaAlg is related to the possibility of mild cross-linking with multivalent cations.^17^ Furthermore, as a natural polysaccharide, NaAlg has been shown to be highly biocompatible and, thus, suitable for medical applications.^18^ However, it can be rather difficult to achieve the desired rheological behavior in solutions based solely on the NaAlg matrix, which leads to the development of combined polymer matrices.

A less common approach to enhancing hydrogel printability involves the use of nanostructured fillers.^19^ This possibility was first described for Laponite, a charged discotic nanoclay, dispersed in poly(ethylene glycol) (PEG) solution.^20^ In an exfoliated state these particles spontaneously form a house-of-cards structure.^21,22^ Laponite, in association with PEG and alginate, provided improved printability and, at higher clay concentrations, elevated recovery after shear.^23^ High shape fidelity of construct was achieved by dÁvila et al., when Laponite–alginate hydrogels were printed.^24^ The capacity to provide rheological tuning to NaAlg solution has also been demonstrated for montmorillonite discotic clay.^25^ Consequently, the principle appears not to be limited to Laponite, but to be a common feature of charged discotic nanoparticles (NPs) suspended in a polymer solution.^22^ The functionality of NPs is largely dependent on their chemical nature, size, aspect ratio, and volume fraction.^26^ Unlike traditional fillers, NPs are capable of inducing significant changes in material properties even at very low concentrations, owing to their large specific surface.^27^ The potential to minimize their presence without compromising the desired effect is particularly advantageous in consideration of medical applications as the risk of immunogenicity is diminished.^4,25,28−31^ The presence of calcium and phosphate ions in scaffolds improves osteoblast adhesion, migration, and differentiation.^32,33^ Additionally, the presence of calcium cations is associated with a decrease of shear stress-induced damage to the cells during bioprinting.^34^

Herein, we focus on the rheological modification of NaAlg solutions via the incorporation of nanoparticulate fillers. We hypothesize that exfoliated layered NPs can serve as efficient rheological modifiers through the formation of a house-of-cards structure. In order to test this hypothesis, layered calcium phenylphosphonate (CaPhP) particles are compared to spherical calcium phosphonate NPs (nanoAp) in terms of rheological modification. Additionally, we address the hydrophobicity of CaPhP with presumed limitation in NaAlg chain intercalation by developing a novel material in which part of the phenyl group is replaced by phosphate. These mixed calcium phenylphosphonate–phosphate (Ca3.1, where 3.1 stand for the ration of reactants during NPs synthesis) nanoplatelets are synthesized, characterized, and exfoliated. This material synthesis is reported here, to the best of our knowledge, for the first time. The rheological and printing evaluation describes the effects of nanofiller shape and chemical composition. Furthermore, the effect of Ca^2+^ dissociation from the NPs is outlined. The printable NaAlg nanocomposites are subsequently used as precursors of hydrogels, which are obtained by ionic cross-linking. Because of the potential to significantly decrease the number of NPs in the material, the hydrogels may find versatile application as 3D printable materials based on natural components. Finally, the materials introduced in this work are proposed and tested as biopolymer-based nanocomposite bioinks.

## Materials and Methods

### Synthesis of Layered Materials

CaPhP: A solution of phenylphosphonic acid (Sigma-Aldrich, St. Louis, MO, p.a., 1.976 g, 1.2 × 10^–2^ mol in 50 mL of distilled water, conductivity <2 μS cm^–1^) was brought to pH 9 by adding concentrated ammonia solution (Honeywell Fluka, Charlotte, NC, p.a., 32%) and then mixed with a solution of CaCl_2_ (Penta, Chrudim, Czechia, p.a.) (1.381 g, 1.2 × 10^–2^ mol in 25 mL of distilled water). The reaction mixture was stirred for 30 min at 25 °C. A white precipitate formed and was collected by filtration, washed with distilled water until neutral pH, and dried in an oven at 80 °C.

Ca3.1: Phenylphosphonic acid (0.79 g, 0.5 × 10^–2^ mol) was dissolved in 50 mL of distilled water (conductivity <2 μS cm^–1^) and mixed with 15 mL of 1 M H_3_PO_4_ (Sigma-Aldrich, St. Louis, MO, p.a., 1.5 × 10^–2^ mol). The pH of the reaction mixture was increased to 9 by a concentrated aqueous ammonia solution. Then 25 mL of CaCl_2_ solution (2.21 g, 2 × 10^–2^ mol) was added under stirring by a magnetic stirrer. The reaction mixture was stirred for 1 h (a) at 25 °C, (b) in an oil bath at 50 °C, and (c) in an ice bath. A white precipitate formed and was collected by filtration, washed with distilled water, and dried in an oven at 80 °C. Other samples were prepared by the same procedure, varying the molar ratio of acids H_2_PhP:H_3_PO_4_ (1:3, 1:1, 3:1, and 0:1), while the molar ratio of P:Ca remained constant (1:1).

Sample characterization: Powder X-ray diffraction (XRD) data were obtained with a D8 Advance diffractometer (Bruker AXS, Karlsruhe, Germany) with Bragg–Brentano θ–θ geometry (40 kV, 30 mA), using Cu Kα radiation, and equipped with a LynxEye detector with a Ni-beta filter. The scan was performed at room temperature from 4° to 90° (2Θ) in 0.01° steps with a counting time of 10 s per step.

Energy-dispersive X-ray (EDX) analysis was performed using a JSM-5500LV electron scanning microscope (JEOL, Tokyo, Japan) equipped with an EDX microanalyzer (detector GRESHAM Sirius 10, IXRF Systems, Austin, TX). The accelerating voltage of the primary electron beam was 20 kV.

Organic elemental analysis (C, H) was performed on a Flash 2000 CHNS elemental analyzer (Thermo Fisher Scientific, Waltham, MA).

### Liquid-Based Exfoliation of Particles

Selection of suitable solvent: 10 mg of powder material was dissolved in 5 mL of solvent (water, isopropyl alcohol, ethylene glycol, or glycerol, all Sigma-Aldrich, St. Louis, MO, p.a.) in a glass vial. The obtained dispersions were treated by ultrasound in an ultrasound bath with frequency 37 kHz for 1 h. Then the sedimentation and presence of Tyndall scattering were evaluated 1 and 24 h after the end of ultrasound treatment.

Preparation of stock dispersions: 200 mg of powder material (CaPhP or Ca3.1) was dispersed in 40 mL of ethylene glycol and treated by a T10 Standard Ultra-Turrax high-shear homogenizer (IKA, Staufen, Germany) equipped with a dispersing tool (S10 D-7G-KS-65) at 13000 rpm for 5 min.

### Ink Preparation for 3D Printing

The proposed 3D printing inks were based on a 3 wt % solution of NaAlg (medium viscosity, Sigma-Aldrich, St. Louis, MO) dissolved in demineralized (DEMI) water (Millipore Q system, Merck, Rathway, NJ). The solutions were prepared by adding the appropriate amount of polymer to DEMI water and dissolving at 50 °C for 18 h in an oven without mixing. This procedure provided a viscous solution, which was mixed with further components.

Layered CaPhP and Ca3.1 particles as well as spherical nanoapatite (nanoAp) particles (<150 nm particle size, Sigma-Aldrich, St. Louis, MO) were used in the form of ethylene glycol dispersions. The concentration of particles in such dispersions was 5 g L^–1^. Also, CaCl_2_ was dissolved in ethylene glycol to provide a solution of the same concentration, i.e., 5 g L^–1^.

The previously described dispersions, as well CaCl_2_ solution, were added to NaAlg solution in concentration of 2 × 10^–6^ particles/CaCl_2_ per 1 mL of NaAlg. The resulting solution was then stirred vigorously with a glass rod in order to obtain a homogeneous mixture. The mixing was typically accompanied by an increase in viscosity and resulted in the pastelike appearance of the materials. Both components were kept at room temperature during the process. These materials were subjected to tests designed to establish 3D printing. In the following text, this stage of materials preparation will be termed “pre-cross-linked inks”.

### Rheological Characterization

Rheological characterization of the pre-cross-linked inks was performed on an MCR 502 rotational rheometer (Anton-Paar, Graz, Austria) at 25 °C, using 25 mm parallel plate geometry. The shear flow behavior measurement occurred when the system was oscillating at constant 10% deformation with angular frequency sweep increasing from 0.1 to 630 rad s^–1^.

Additionally, the inks were subjected to cyclic shear stress in order to characterize them from the 3D printing point of view. The measurement was conducted in oscillation mode, switching between low (3 rad s^–1^) and high (100 rad s^–1^) angular frequency. Both low and high angular frequencies were always held for 50 s, and the transition was instant.

### 3D Printing Experiments

Microextrusion 3D printing experiments were performed on pre-cross-linked inks with a BioX bioprinter (Cellink, Gothenburg, Sweden) with the following specifications: polypropylene conical nozzle of 0.41 mm inlet diameter and 0.26 mm outlet diameter, 3 mL polypropylene syringe, microextrusion syringe pump printhead, and microscope glass slide printbed. The printhead speed was 2 mm s^–1^, and the extrusion rate was varied between 1 and 1.5 μL s^–1^ according to each material’s specific behavior. During printing, both printhead and printbed were kept at 25 °C.

The printing ability of the pre-cross-linked inks was evaluated by a method derived from the filament fusion test.^35^ A zigzag pattern was printed, with the distance between the strands increasing at increments of 0.1 mm. This increment was chosen with consideration for the high swelling of the inks during microextrusion. Images of the printed patterns were taken using a Dino-Lite AM4815ZT optical microscope (AnMo Electronics Corporation, Taipei, Taiwan) and evaluated with the aid of the ImageJ software (v1.5, Wayne Rasband, National Institutes of Health, Bethesda, MD). Three characteristics were obtained from the test: strand thickness, partial fusion distance, and complete separation distance.

### Compressive Strength Testing

Young’s modulus in compression was measured on 3D printed cylinders (10 mm × 10 mm). Prior to compression testing, stabilization of the materials was performed in the following way: The structures obtained from pre-cross-linked inks were immersed in a 2 wt % aqueous solution of CaCl_2_ for 30 min. The CaCl_2_ solution was kept at 25 °C, and no mixing was applied during the final cross-linking. The samples subjected to this treatment will be termed “fully cross-linked hydrogels” in the following text. An Instron 3345 device (Instron, Norwood, MA) with a 100 N force transducer was used for compressive strength analysis. The measurement occurred at constant deformation rate of 1 mm min^–1^.

### Morphological Analysis

Scanning electron microscopy (SEM) imaging was used for observation of the composite hydrogels. Vertical sections of freeze-dried samples of the fully cross-linked hydrogels were observed. The SEM analysis was done using the Phenom Pro instrument (Thermo Fisher Scientific, Waltham, MA) at an accelerating voltage 10 kV. The samples were sputtered with a gold/palladium layer prior to imaging.

Atomic force microscopy (AFM) was used to measure the topological profile of the exfoliated NPs and to determine the structure and morphology of the hydrogel samples. Measurement was performed at room temperature on a Dimension ICON atomic force microscope (Bruker, Karlsruhe, Germany) in peak force mode with a ScanAsyst tip. Samples of exfoliated particles were centrifuged for 5 min at 6000 rpm, spin-coated on atomically flat mica substrate, and dried at 60 °C. The morphology of the hydrogels was measured in sections of native hydrogel samples. The samples were prepared by cryomicrotome cutting by a diamond knife (Micro Star) at −80 °C. The primary particle size distribution was analyzed by the SW^36^ in accordance with refs (37−39). The sizes are presented as the histograms of the equivalent circles’ diameters.

X-ray computed microtomography (CT) analysis of the printed structures and their porosity was performed with the help of the SkyScan 1174 device (Bruker, New York, NY). The device used an X-ray source (voltage 20–50 kV, maximum power of 40 W) and an X-ray detector. The CCD 1.3 Mpix was coupled to a scintillator by a lens with 1:6 zoom range. The projection images were recorded at angular increments of 0.5° or 1° using tube voltage and tube current of 35 kV and 585 μA, respectively. The exposure time was set to 15 s, and no filter was used. The 3D reconstructions and the analysis of the surface, volume, and porosity of the structures were performed via built-in CT image analysis software (v1.16.4.1, Bruker). The results, in terms of images with different X-ray adsorption, and 3D models were exported from Data Viewer and CTVox v1.16.4.1 software (Bruker). Prior to CT characterization, the printed hydrogels were placed in a closed sample holder with increased humidity in order to prevent drying out during the measurement process. The porosity was calculated only for a central part of the sample (a cylindrical area of 500 mm × 400 mm) in order to exclude the irregularities of the 3D surface from CT.

### *In Vitro* Cytotoxicity Testing

For the cytotoxicity study, 3T3 fibroblasts (DSMZ, Braunschweig, Germany) were used. These were cultured in a CO_2_ incubator in Dulbecco’s Modified Eagle Medium (DMEM) containing 10% fetal bovine serum (FBS), streptomycin, penicillin, and l-glutamine, which were all purchased from Gibco (Life Technologies, Grand Island, NY).

An MTT assay was performed using 3-(4,5-dimethyldiazol-2-yl)-2,5-diphenyltetrazolium bromide (MTT) purchased from Merck (Rahway, NJ). MTT at concentration 0.5 mg mL^–1^ was diluted in full growth media and sterile filtered through a 0.22 μm filter (TPP Techno Plastic Products AG, Trasadingen, Switzerland). After 24 h incubation of cells with testing samples, the medium in 96-well TC plates was replaced with 100 μL of MTT solution and incubated for 3 h. Then the MTT solution was removed, and 100 μL of dimethyl sulfoxide was added to the wells. Absorbance was determined at 595 nm using a Multiskan FC photometer (Thermo Fisher Scientific, Waltham, MA).

NPs for MTT cytotoxicity assay were prepared as follows: 10 mg mL^–1^ of NPs was resuspended in ethylene glycol, and ultrasound was applied for 1 h. Then the full growth medium (DMEM, 10% FBS) was used to prepare NP dilutions in the range 10^–2^–10^–9^ mg mL^–1^. 3T3 fibroblasts were seeded on 96-well TC plates at a density of 5000 cells per well and incubated overnight before NPs were applied to the cells. The cells were treated with NPs for 24 h. Afterward, the MTT assay was performed as described above.

The cytotoxicity of hydrogels was determined using hydrogel extracts and direct contact according to ISO10993-12.

### Bioprinting

Bioprinting was performed with BALB mouse fibroblast cell lines. The cells were cultivated in DMEM (Sigma-Aldrich, St. Louis, MO) containing 10% fetal calf serum (FCS, Biosell, Nuremberg, Germany), 1% GlutaMAX (Gibco, Life Technologies, Grand Island, NY), and 0.1% gentamycin sulfate (Sigma-Aldrich) at 37 °C in a humidified incubator (95% relative humidity, 5% CO_2_; Thermo Fisher Scientific, Waltham, MA). The cells were split using 0.05% trypsin (Sigma-Aldrich).

Bioinks were obtained by preparing pre-cross-linked inks containing either CaPhP or Ca3.1 according to the procedure described in the section Ink Preparation for 3D Printing and adding the cell suspension in phosphate buffered saline (PBS, pH 7.4) in 100 μL/1 mL v/v ratio, followed by thorough stirring. The bioinks were loaded into cartridges and printed by pneumatic extrusion printhead, using the 3D Discovery Bioplotter (RegenHU, Villaz-Saint-Pierre, Switzerland). The printing was done through conical nozzles or cylindrical needles of 0.52, 0.41, or 0.21 mm diameter. A printing model of a simple 1 cm × 1 cm grid was chosen. The printing pressure varied according to the bioink’s properties.

Stabilization of the inks was done by immersing the prints in 0.1 wt % FeCl_3_ solution in PBS for 2 h. Following this, live/dead staining was performed. The staining solution was prepared as follows: 2 μL of ethidium homodimer I (dead stain) and 2 μL of calcein acetoxymethyl ester (live stain) were diluted in 10 mL of PBS. The staining solution was poured over the prints in a sufficient amount and left in an incubator for 1 h to allow full diffusion of the staining solution through the material. The stained cells were visualized in a DMI6000 fluorescence microscope (Leica, Wetzlar, Germany). The percentage of living cells was determined in three different places in the printed structure, and the results were averaged.

**Figure 1:**
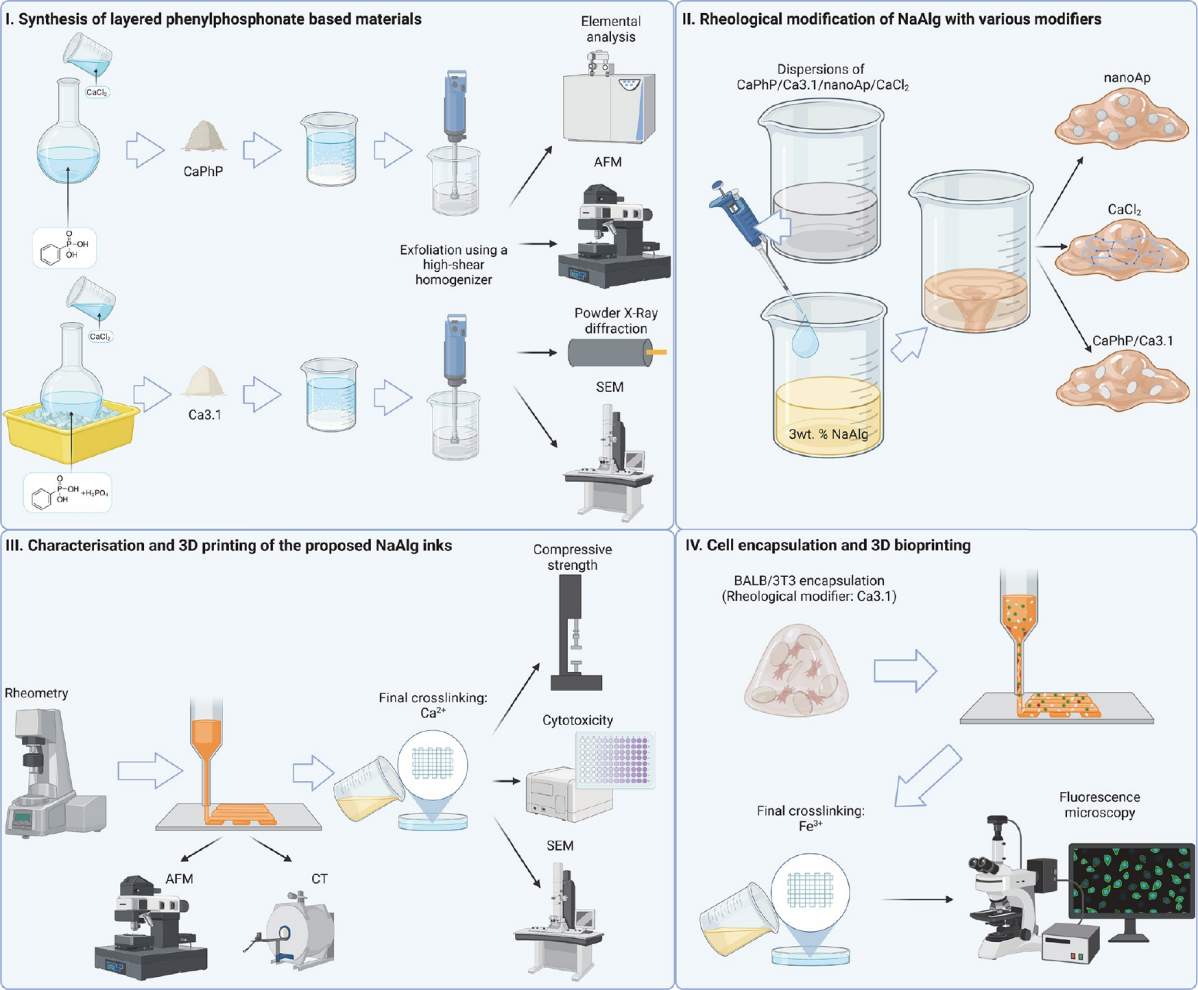
Schematic representation of the experimental workflow (created with BioRender.com).

## Results and Discussion

### Synthesis and Characterization of New Calcium Phenylphosphonate–Phosphate

The synthesis and exfoliation of CaPhP, as well as its use for preparation of polymer–inorganic nanocomposites, were described in our previous work.^41^ To increase possible interactions and compatibility with water-based systems, such as hydrogels, new material was synthesized where part of the phenyl groups was replaced by phosphate, followed by characterization, exfoliation, and optimization of the nanocomposites with NaAlg solution for 3D printing.

The synthesis of mixed calcium phosphonate–phosphate follows the same route as preparation of CaPhP which has been described in our previous works.^40,41^ Whereas for obtaining pure CaPhP, keeping the pH > 9 is the only necessary condition, for material with combined phosphonate and phosphate groups there are other important factors influencing the properties of the final product. The molar ratio of the reacting acids determines not only the composition but also the basal spacing of the final product. Different molar ratios were tested (see Table 1). Despite the high affinity of phosphonate moieties to Ca^2+^ ions,^42^ it was observed that phosphoric acid forms structures with Ca^2+^ more easily than phosphonate acid. Where H_3_PO_4_ excess occurs in the reaction mixture (H_2_PhP:H_3_PO_4_ ratio 1:3), only the layered structure of brushite is formed, which does not contain any phenylphosphonate anions (see Figure S1 in the Supporting Information). When the H_2_PhP:H_3_PO_4_ ratios 1:1 or 3:1 were used, layered phases with a basal spacing of about 15 Å were formed, together with some hydroxylapatite. The amount of hydroxylapatite was lower for the 3:1 acid ratio. As temperature plays a significant role in hydroxylapatite formation,^43^ change of reaction temperature, either heating to 50 °C or cooling in an ice bath, was proposed to support phenylphosphonate–phosphate formation over hydroxylapatite precipitation. It was observed that both heating and cooling of the reaction can successfully suppress hydroxylapatite formation, leading to pure Ca3.1 in the case of acid ratio 3:1 (see Figure S2). However, in the case of acid ratio 1:1, hydroxylapatite forms in significant quantities regardless of the reaction temperature. Additionally, the reaction mixture tends to thicken while heating, which causes difficulties with stirring. This problem is not present in cooled reaction conditions. Thus, the sample (Ca3.1) prepared from a reaction mixture of 3:1 acid ratio, which was cooled in an ice bath, was selected for exfoliation and application in 3D printing ink. The purity of the product was determined by XRD as well as by basal spacing, which is given in Table 1. The diffraction pattern of this compound can be indexed (see Table S1) in a monoclinic system with lattice parameters shown in Table 2. Its chemical composition was verified by elemental analysis (found: C = 22.47%, H = 2.92%; calculated: C = 22.96%, H = 3.02%), and the molar ratio P:Ca 1:1 was confirmed by EDX. The morphology of the resulting particles was determined by SEM imaging (see Figure S3).

**Table 1:** Influence of the Molar Ratio of Acids on the Obtained Phases

sample	molar ratio H_2_PhP:H_3_PO_4_	basal spacing (Å)	composition
CaPhP	1:0	15.0	Ca(C_6_H_5_PO_4_)·2H_2_O
Ca3.1	3:1	15.0	Ca(C_6_H_5_PO_3_)_0.62_(HPO_4_)_0.38_·1.18H_2_O
Ca1.1	1:1	15.2	contains Ca_5_(PO_4_)_3_(OH)
Ca1.3	1:3	7.6	HCa(PO_4_)(H_2_O)_2_
Ca0.1	0:1	7.6	HCa(PO_4_)(H_2_O)_2_

**Table 2:** Crystallographic Data of the Prepared Ca3.1 Material

material	Ca3.1
crystal system	monoclinic
*a*	19.3258 ± 0.0003 Å
*b*	11.0287 ± 0.0003 Å
*c*	5.7158 ± 0.0001 Å
β	129.055 ± 0.004°

### Liquid-Based Exfoliation of Particles

Successful liquid-based exfoliation depends on suitable solvent selection. An appropriate solvent that possesses good compatibility with the material submitted to exfoliation can act as an exfoliating as well as a stabilizing agent for nanoplatelets formed during the process.^44^ The suitability of the combination of material and solvent can be tested by the simple procedure described by Kopecká et al.,^41^ in which a small amount of material in solvent is treated by ultrasound for 1 h. The stability of the obtained dispersion is observed and the presence of NPs is verified by Tyndall scattering.^45^ In the present work, four solvents were tested: water, isopropyl alcohol, ethylene glycol, and glycerol. These solvents were chosen with respect to their polarity, promising good compatibility with Ca3.1 nanoplatelets. The second parameter in selection of a solvent was its miscibility with water, as the dispersions of exfoliated particles are intended as components of hydrogel-based 3D printing ink.

The best results were obtained for isopropyl alcohol, where no sedimentation occurred. Ethylene glycol dispersions also showed good stability, with only small amounts of material, which settled down. In the case of glycerol, significant sedimentation was observed, although Tyndall scattering was still present. Distilled water did not facilitate any stability in the dispersion, as no Tyndall scattering could be observed after 1 day of sedimentation. This means that the initial assumption that the presence of phosphate groups enhances compatibility with water was not confirmed. However, the potential to perform liquid-based exfoliation in ethylene glycol provides a material highly miscible with water-based systems, with low cytotoxicity.^46^ Therefore, we have successfully obtained a material suitable for mixing with aqueous biopolymer solution.

**Figure 2:**
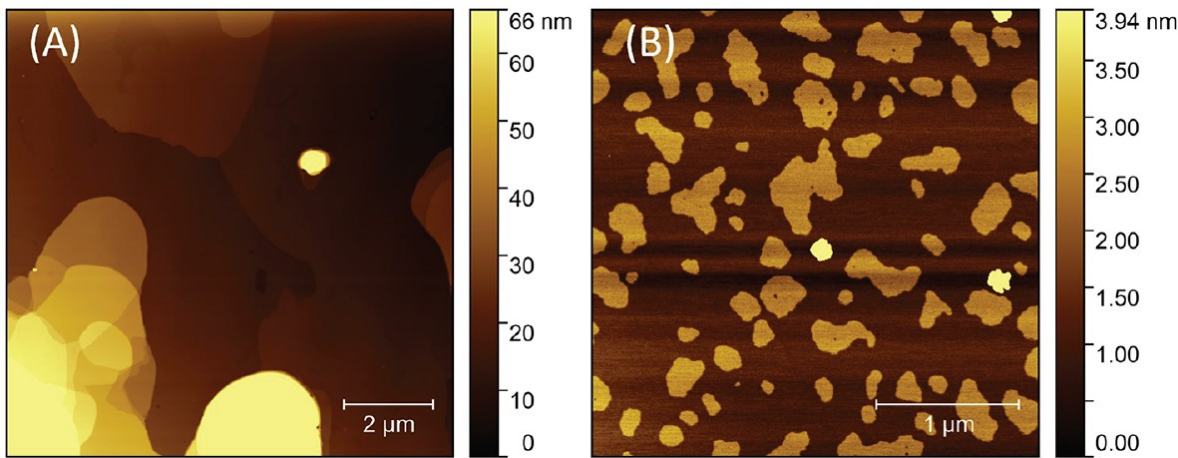
Atomic force microscopy scans of exfoliated particles. Nanoplatelets of (A) Ca3.1 exhibit greater lateral dimensions than nanoplatelets of (B) CaPhP.

Furthermore, the morphology of the Ca3.1 nanoplatelets was characterized and compared to previously synthesized CaPhP.^40^ When liquid-based exfoliation was performed by high shear homogenizer in the same conditions for CaPhP and Ca3.1, the mixed structure resulted in nanoplatelets with larger lateral dimensions, as determined by AFM (Figure 2), which showed Ca3.1 particles typically in the range of 3.2–3.6 nm thickness with lateral dimensions varying from 2 to 10 μm after the exfoliation process. It is assumed that the overlaps are created upon drying, but in the dispersion the individual lamellae are available.^45^ The number-based histograms illustrate the decrease of the lateral size for CaPhP nanoplates (the most-populated equivalent diameter ca. 200 nm) in comparison to Ca3.1 (1300 nm) (Figure S4).

### Rheological Characterization of NaAlg Nanocomposites

3D printing via microextrusion is a technological process, typically requiring non-Newtonian liquids to achieve the desired results. In general, low viscosity is advantageous during extrusion in order to minimize energy consumption and the mechanical stress applied to the material. However, as the material is placed freely on the printbed, high viscosity is required in order to maintain the cylindrical shape of the filament. Hence, the ideal rheological behavior of a 3D ink is shear thinning.^47^ Here, we compare the ability of layered calcium phosphonate particles to rheologically tune NaAlg solutions with that of spherical nanoAp particles.

**Figure 3:**
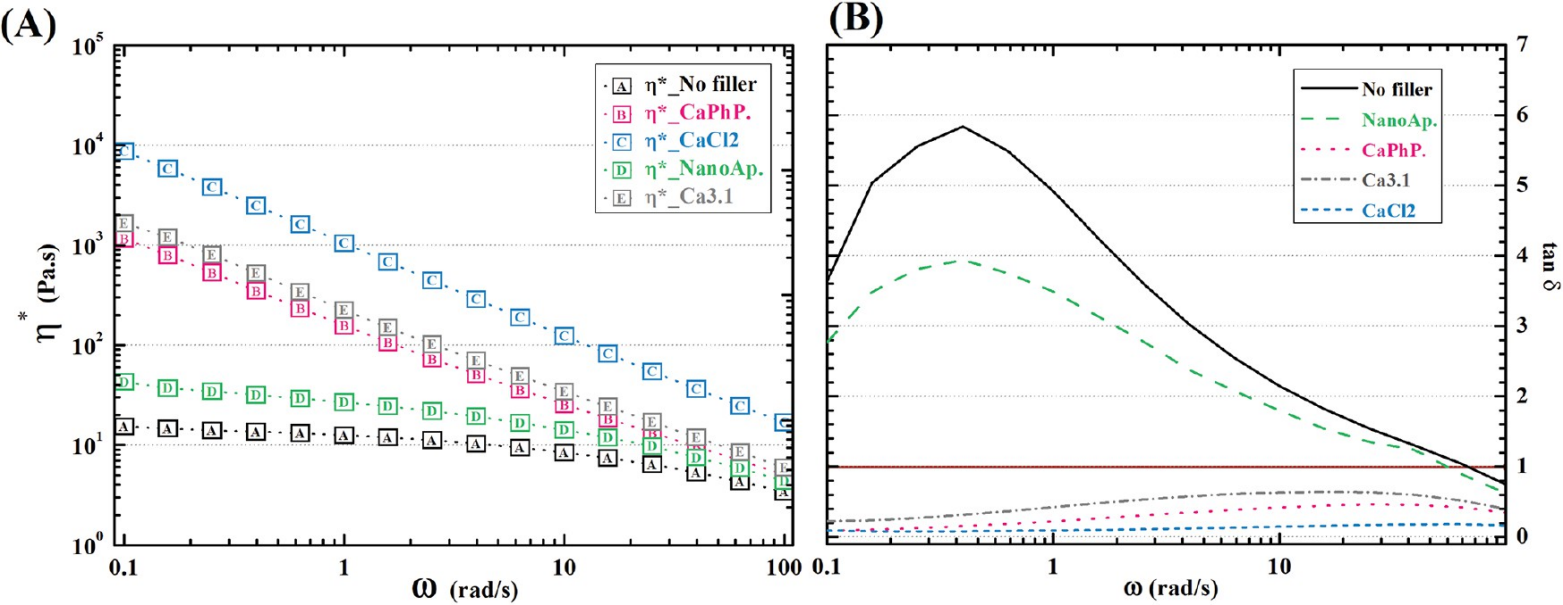
Dependence of (A) viscosity and (B) damping factor of pre-cross-linked inks on angular frequency.

The pre-cross-linked inks displayed clear shear thinning in the range of 10^–1^–10^2^ rad s^–1^ (see Figure 3A). The microextrusion process typically corresponds to angular frequencies of 10^2^ rad s^–1^; therefore, the selected range is sufficient to satisfy characterization requirements.^47^ Three of the assessed inks showed initial viscosity in the range of 10^3^–10^4^ Pa·s, while all the compositions reached viscosities of 10^1^ Pa·s upon approaching angular frequencies of 10^2^ rad s^–1^. The spherical nanoAp made virtually no difference to a pure 3% solution of NaAlg. Therefore, these particles are not suitable as a viscosity-modifying additive. Furthermore, the non-Newtonian tendency is rather weak in the case of these two compositions, and the initial viscosity is in the range of 10^1^ Pa·s, which would presumably lead to high spreading of the filament upon deposition during printing.

This assumption is further supported by the damping factor, i.e., loss-to-storage modulus ratio (Figure 3B). Where the viscous response prevails, the material appears to be liquid; otherwise, the material is solid. Therefore, the damping factor gives an overall idea of the material’s apparent behavior. A damping factor above 1, which was measured in the case of pure NaAlg as well as in nanoAp-modified NaAlg, determined these inks’ behavior as liquidlike in the whole range of angular frequencies studied here. Conversely, the other viscosity-modifying additives caused the NaAlg to appear as a solid throughout the measurement. It can be assumed that the nanofillers dissociate some Ca^2+^ ions, which are known to cross-link polyanionic polymers, such as NaAlg.^48^ In order to assess the influence of the free calcium ions on the rheology of NaAlg solution, one sample contained no nanoparticulate fillers, but only free Ca^2+^ ions. This sample displayed the lowest damping factor, determining that the presence of multivalent ions is a significant factor in the thickening of NaAlg solution. However, the high prevalence of storage over loss modulus in this case may cause further problems in 3D printing accuracy due to the phenomenon of overgelation.^49^

**Figure 4:**
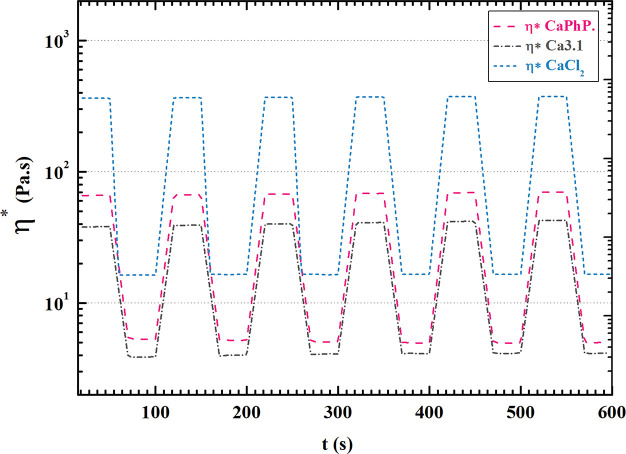
Cyclic shearing simulation of the microextrusion process.

3D printing via microextrusion involves two main shear states of the material: (a) extrusion, during which the material is subjected to shear rates of 10^2^ rad s^–1^ and (b) deposition, upon which the shear stress suddenly drops to zero. In order to more closely assess the suitability of the nanocomposite hydrogels for microextrusion 3D printing, the three selected inks were subjected to cyclic shear stress rapidly oscillating between high (extrusion) and low (deposition) shear stress. Figure 4 clearly shows that the rapid change in shear stress induces a change of viscosity which occurs within 10 s for any given rheological modifier. Furthermore, the hysteresis between cycles is minimal. These qualities are essential for a successful microextrusion process.^50^

**Figure 5:**
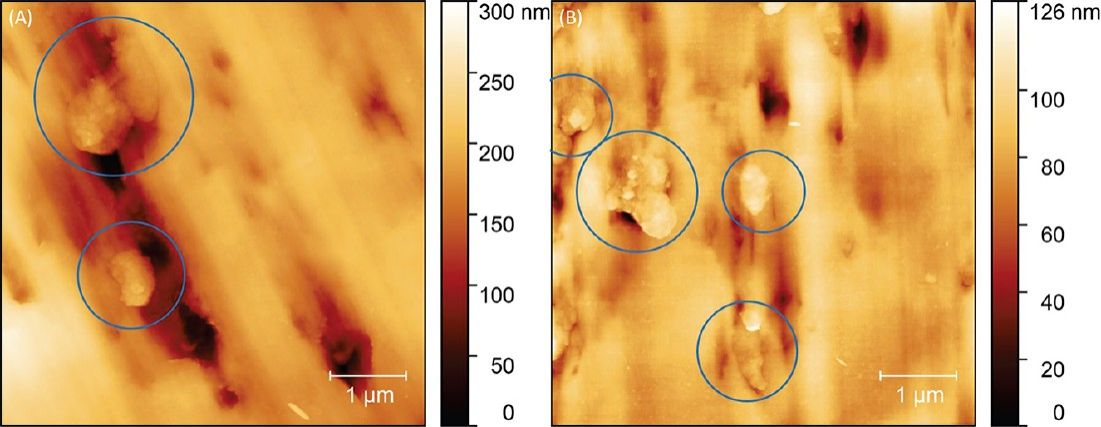
Atomic force microscopy topology scans of the section of the modified NaAlg hydrogel samples by (A) Ca3.1 and (B) CaPhP, where randomly oriented aggregates of nanoplatelets are observed.

It is also clear that there is a large difference in the magnitude of the change, with the largest occurring with addition of CaCl_2_, followed by CaPhP, and Ca3.1. While a large difference in viscosity is generally beneficial to microextrusion printing, the viscosity values in the case of high shear stress should be noted. In that situation it is clear that CaPhP and Ca3.1 containing NaAlg inks reach the same orders of magnitude of viscosity as pure NaAlg solution (Figure 3A) at 10^2^ rad s^–1^, while the viscosity of CaCl_2_ is 1 order higher in comparison. This may suggest a certain percentage of permanent cross-linking being present in the latter case. This would result in high irregularity of the printed strand during microextrusion.^49,51^ It could lead to higher occurrence of defects and result in overall mechanical weakening of the printed object. However, the shear-thinning behavior of the CaPhP- and Ca3.1-modified NaAlg pre-cross-linked inks could be the result of a flow-induced orientation of NPs. This phenomenon has been reported for Laponite filler.^23,50^ Due to the presence of ionic moieties on the surface of the particles, it is reasonable to assume the occurrence of a house-of-cards structure due to the particles’ surface saturation by guluronic acid units of NaAlg. Thus, the rheological behavior of the presented pre-cross-linked inks could be the result of analogous phenomena.^23^ Additionally, it was attempted to observe the nanoplatelets positioning in the pre-cross-linked inks by AFM. As can be seen in Figure 5, it was possible to observe randomly arranged particles in the cross section, which is in agreement with the hypothesis of the house-of-cards structure formation. However, it should be noted that AFM is in principle a surface measurement technique, and it may not reflect the positioning of the platelets in the bulk. Furthermore, the dimensions of the observed particles are larger compared to Figure 2, possibly suggesting incomplete exfoliation of the nanoplatelets. Despite this drawback, the CaPhP and Ca3.1 fillers facilitate significant rheological modification of the NaAlg solution.

When all the parameters are taken into account, the rheological evaluation favors the additive as the most suitable for 3D printing via microextrusion, in terms of viscosity modification of NaAlg. These assumptions are further tested by experimental microextrusion.

### 3D Printing

**Figure 6:**
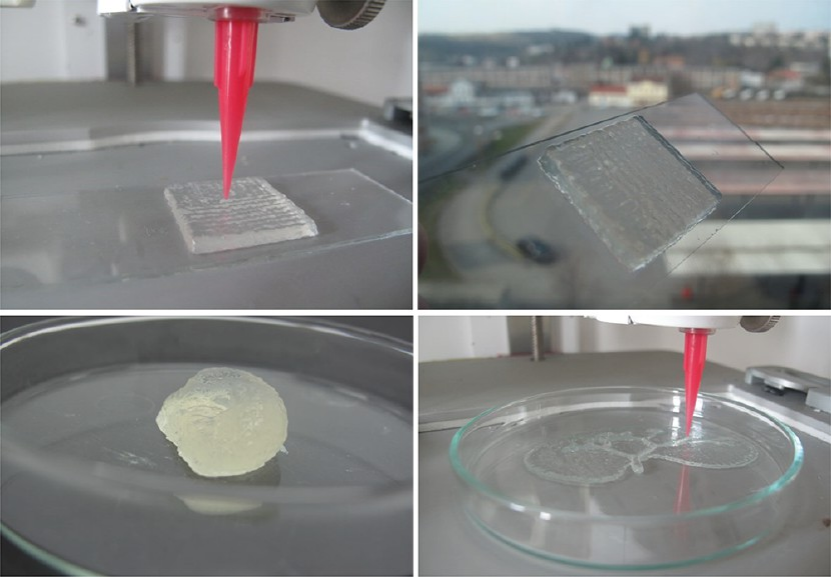
Structures obtained by 3D printing using either CaPhP- or Ca3.1-modified NaAlg.

The main advantage of manufacturing via 3D printing is the ability to accurately place the material and all its components in the desired shape and, thus, form highly homogeneous and precise structures. Printing precision depends on material characteristics as well as processing parameters.^52^ It has been shown above that the inks tested display similar rheological behavior at high shear rate 4. Therefore, it is possible to obtain good printing results, as demonstrated by the printing of several structures, which can be seen in Figure 6.

**Figure 7:**
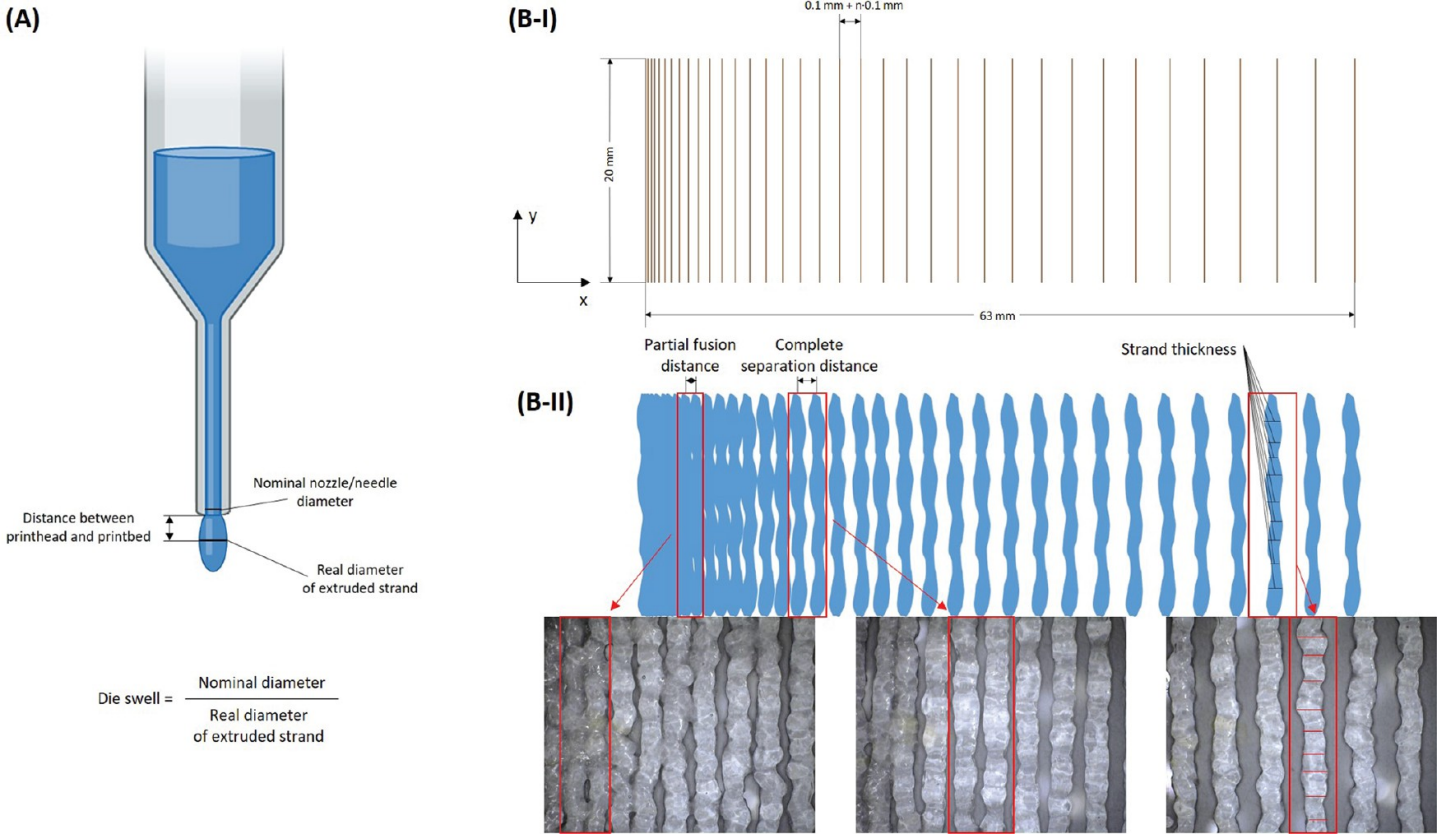
Schematic representation of evaluation of printing precision characteristics: (A) die swell, (B-I) filament fusion test printing model, and (B-II) example of filament fusion test evaluation.

The assessment of shape fidelity proposed by Ribeiro et al.^35^ describes a filament fusion test method. This method makes readily available three parameters connected to printing precision. The current study is focused on strand thickness, partial fusion distance, and complete separation distance. The evaluation methodology is schematically described in Figure 7. Table 3 presents the obtained data.

**Table 3:** Printing Precision Characteristics of the Examined Pre-Cross-Linked Inks

	type of filler	die swell	strand thickness (mm)	partial fusion distance (mm)	complete separation distance (mm)
nozzle diameter 0.26 mm	CaCl_2_	3.0 ± 0.5	0.33 ± 0.08	1.7	1.9
	nanoAp	3.2 ± 0.2	0.56 ± 0.09	2.2	2.5
	Ca3.1	1.4 ± 0.2	0.45 ± 0.05	1.4	1.8
	CaPhP	2.3 ± 0.2	0.39 ± 0.06	1.4	1.8
nozzle diameter 0.42 mm	CaCl_2_	2.2 ± 0.4	0.6 ± 0.1	1.7	1.9
	nanoAp	2.2 ± 0.4	0.8 ± 0.1	2.5	2.9
	Ca3.1	1.42 ± 0.12	0.51 ± 0.07	1.5	1.9
	CaPhP	2.0 ± 0.2	0.45 ± 0.08	1.5	1.9

In an ideal 3D printing ink, the strand thickness matches the nozzle diameter. However, hydrogel inks typically provide thicker strands than the ideal case. In order to provide complex characterization of the proposed inks, the die swell of material suspended from the nozzle and the strand thickness obtained on the printbed were measured. The strand thickness can be easily related to the ideal ink by the so-called swelling ratio, or the ratio of strand diameter to nozzle diameter. A decrease in nozzle diameter increases the shear stress placed upon the material during flow. Die swell, i.e., the ratio of real strand diameter of suspended material at the exit of the nozzle to the nominal nozzle diameter, is in direct connection to the normal stresses induced during shear flow of the material.^53^ It is clear from the data in Table 3 that lower nozzle diameter induces higher normal stresses, causing more significant die swell. Furthermore, the pre-cross-linked ink containing Ca3.1 particles appears to be the least susceptible to the influence of normal stresses. The presence of fillers influences the rheological behavior of polymeric systems. There are several characteristics that need to be taken into account, mainly the concentration and geometry of the filler.^54^ It has been found that the addition of solid particles in low concentrations generally decreases die swell. This effect is the most pronounced for fibers and flakes.^55^ This is consistent with the findings of the current study, as the lowest die swell was observed for layered nanofillers—CaPhP and Ca3.1—compared to using spherical nanoAp or no filler.

**Figure 8:**
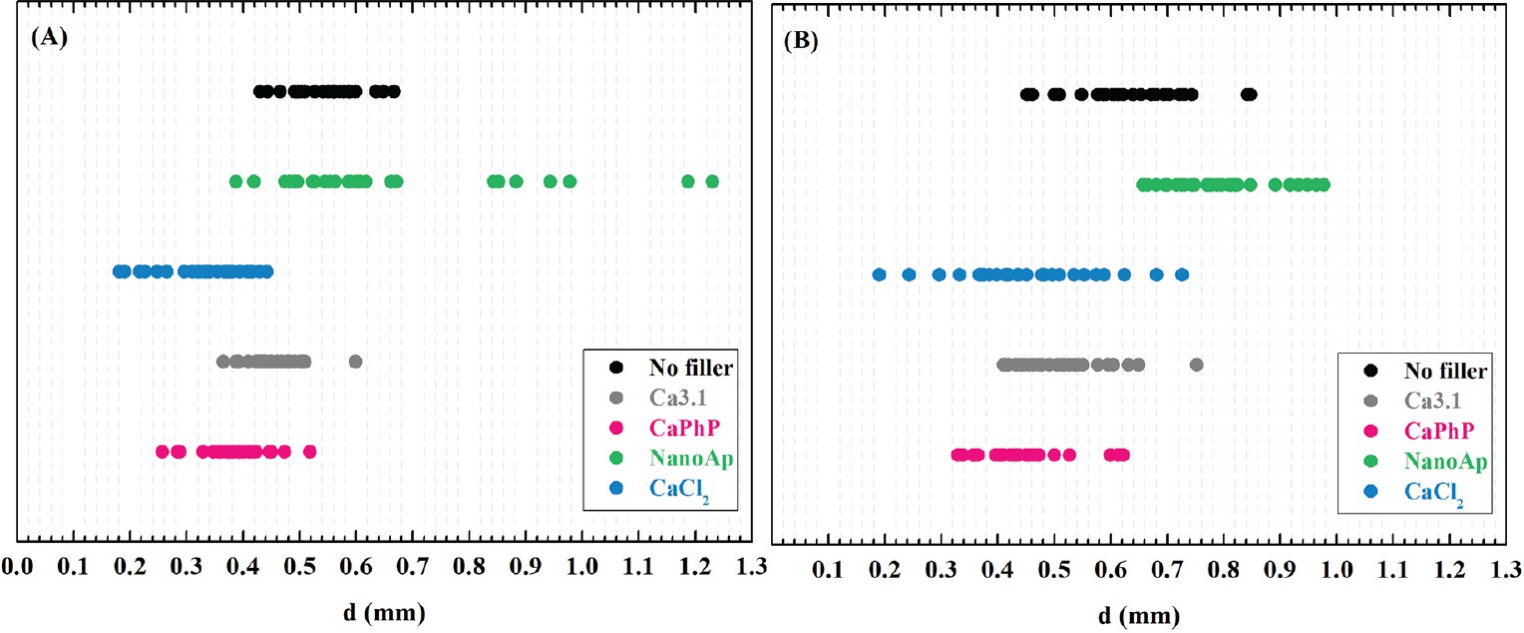
Strand diameter variation diagram: (A) 0.21 mm nozzle; (B) 0.42 mm nozzle.

However, strand thickness does not follow the same trend as die swell. As is apparent from Table 3, Ca3.1 filler leads to higher strand thickness than CaPhP, despite lower die swell. It is possible to conclude that material spreading due to lower viscosity in the steady state (see Figure 4) plays a considerable role. The results for NaAlg pre-cross-linked ink containing only CaCl_2_ (Figure 8) suggest even thinning of the material upon its placement on the printbed. This can be caused by the elongation of the material due to the constant movement of the printhead. The high viscosity of this ink at steady state prevents any material spreading.

The partial fusion distance and complete separation distance are parameters characterizing the nonideality of an ink by taking into account not only the mean strand thickness but also its fluctuations throughout the printing process. Under the assumption of well-designed process parameters in terms of avoiding turbulent flows in the channel, these fluctuations may be caused by the accumulation of stress due to the need to overcome material adhesion to the flow channel walls and also by inhomogeneities, such as improperly dispersed modifier particles and air bubbles. The fluctuations typically occur in the case of overgelation,^49^ when the extreme rigidity of the material prevents smooth flow. It is evident that though a thinner nozzle leads to higher swelling of the strand, the strand thickness distribution is narrower than in the case of a thicker nozzle (Figure 8). The large variations in strand diameter are particularly prominent when CaCl_2_ is incorporated, on account of the overgelation phenomenon. Also, the liquid character of nanoAp-modified pre-cross-linked ink causes high spreading of the material, leading to great unevenness in the strand, which is intensified by greater die swell when a narrow nozzle is used. It can be assumed that the higher shear stress induced by a lower diameter of flow geometry contributes to the elimination of fluctuations in the flow of the material. It is therefore possible to obtain more precise structures with narrow nozzles than with wide ones when die swell is taken into account.

### Morphology of Hydrogels

The advantageous rheological characteristics obtained by the incorporation of layered CaPhP and Ca3.1 in NaAlg solution and satisfactory printing precision allow the printing of constructs consisting of several layers of material (up to 25 layers). However, in order to achieve sufficient long-term stability of the hydrogel and increase its usability in further applications, a secondary form of cross-linking is needed. Because of the polyanionic character of NaAlg, it is readily cross-linkable by the simple addition of multivalent ions. In order to maintain the nontoxicity of the scaffold as well as the process, a 2 wt % solution of CaCl_2_ was chosen as the source of multivalent ions. To assess the influence of ionic cross-linking on the morphology of printed constructs, SEM and CT analyses were applied.

**Figure 9:**
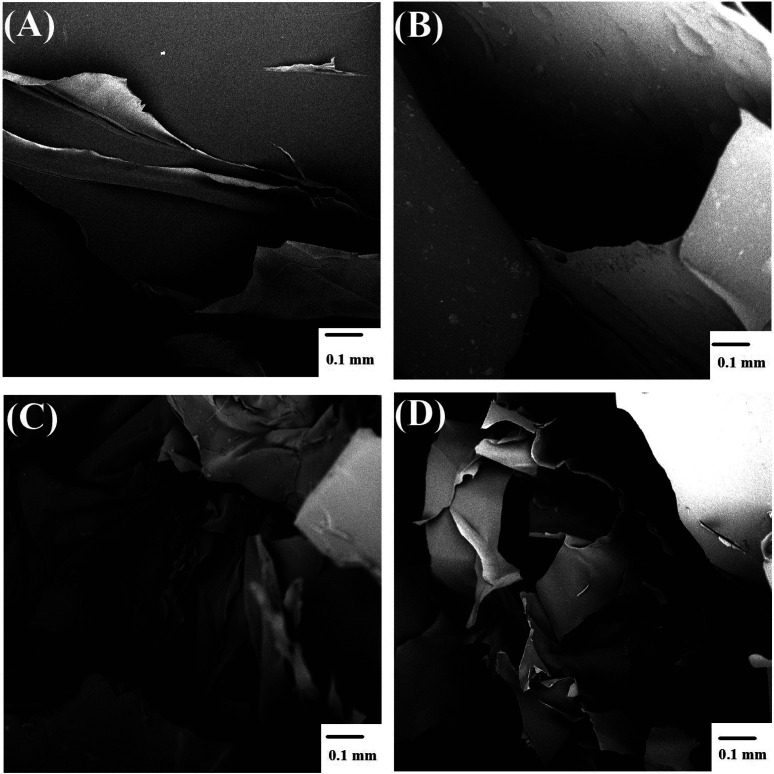
Scanning electron microscopy micrographs of cross-section cuts of freeze-dried hydrogels: (A) pure NaAlg, (B) nanoAp filler, (C) Ca3.1 filler, and (D) CaPhP filler.

Scanning electron microscopy imaging of freeze-dried hydrogels provided an insight into their inner structure. The hydrogels contained large pores, and their structure appeared to consist of laminated layers (see Figure 9).

**Figure 10:**
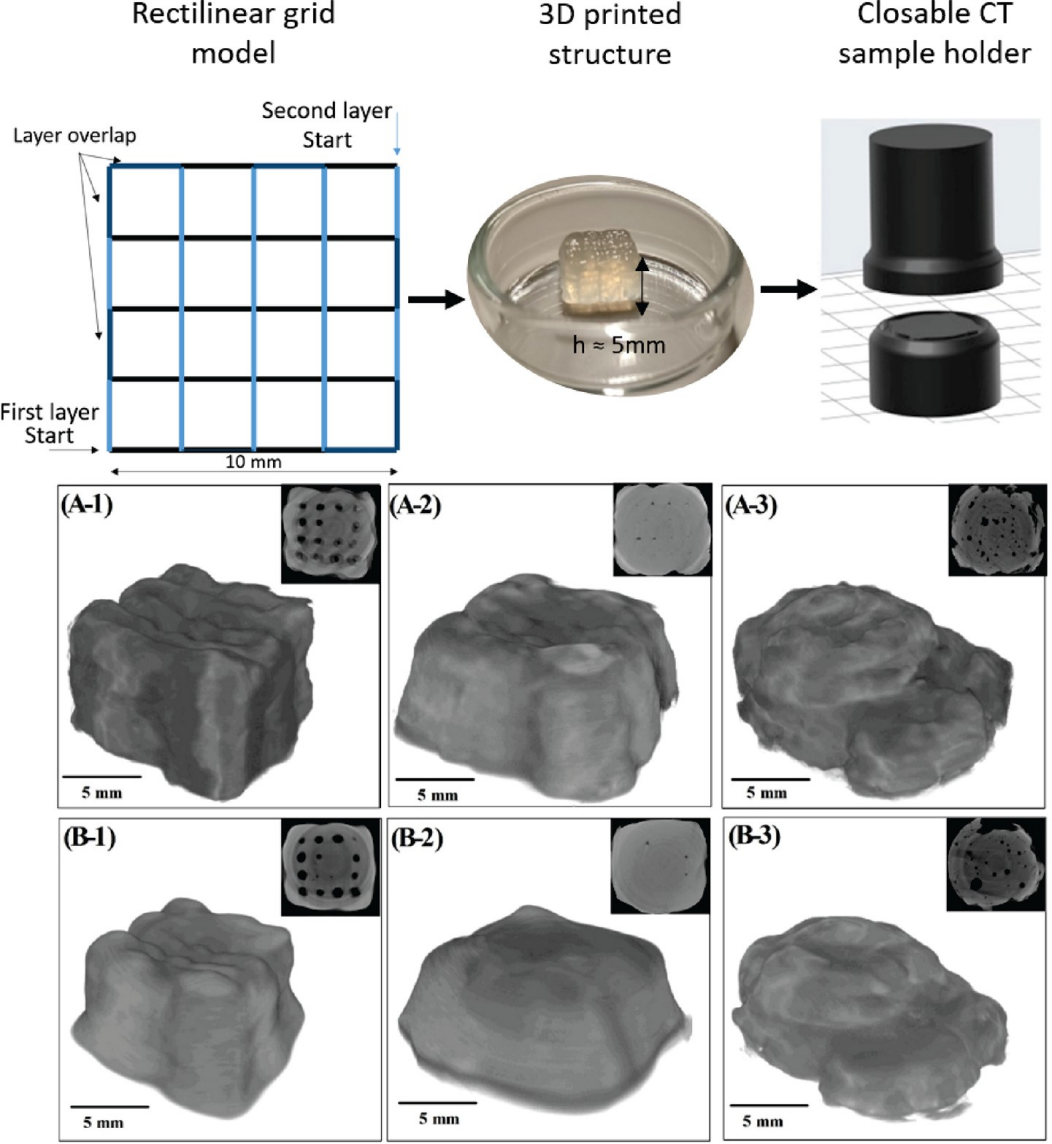
X-ray computed microtomography reconstruction of 3D printed NaAlg structures: (A) pre-cross-linked ink structures, (B) fully cross-linked hydrogel structures, (1) CaPhP filler, (2) Ca3.1 filler, and (3) CaCl_2_ substitute for filler.

CT measurement was used to characterize the overall shape and porosity of the printed constructs (multilayered grids). As seen in Figure 10, the best printing precision was achieved with CaPhP rheological modifier. Furthermore, it is apparent that the printed structures lose some shape fidelity and shrink in volume when ionic cross-linking is applied. The porosity present in the 3D structure due to fabrication by microextrusion was also evaluated from CT data. While CaPhP and CaCl_2_ resulted in open porosity of approximately 25%, Ca3.1 provided open porosity below 0.2% and no closed porosity (see Table 4). This is consistent with the comparatively higher spreading of the material during printing, which was discussed earlier. In all cases, the number of pores slightly decreased with the application of ionic cross-linking. Additionally, open porosity increased in the case of CaPhP- and CaCl_2_-modified NaAlg samples. It can be assumed that the cross-linking causes the structure to collapse, leading to overall shrinkage, partial collapse of the structure, and the joining of pores in close proximity. This results in fewer pores with larger areas, increasing porosity. It can be speculated that the described differences between agents for rheological modification result from their different chemical natures. These nuances ought to alter the material’s surface energy, for both the organophosphonate particles and the composite ink. The higher surface energy of Ca3.1 containing NaAlg may explain part of the discrepancy in the behavior of CaPhP- and Ca3.1-filled hydrogels. However, this assumption needs further evaluation.

**Table 4:** Analysis of Printing Induced Porosity of Hydrogel Scaffolds before and after Ionic Cross-Linking

	open porosity (%)		closed porosity (%)		number of pores	
ionic cross-linking	before	after	before	after	before	after
CaPhP	25.43	25.85	0.61	0.10	25	22
Ca3.1	0.13	0.01	0.01	0.01	5	3
CaCl_2_	23.89	25.85	0.01	0.12	35	22

### Compressive Strength

**Figure 11:**
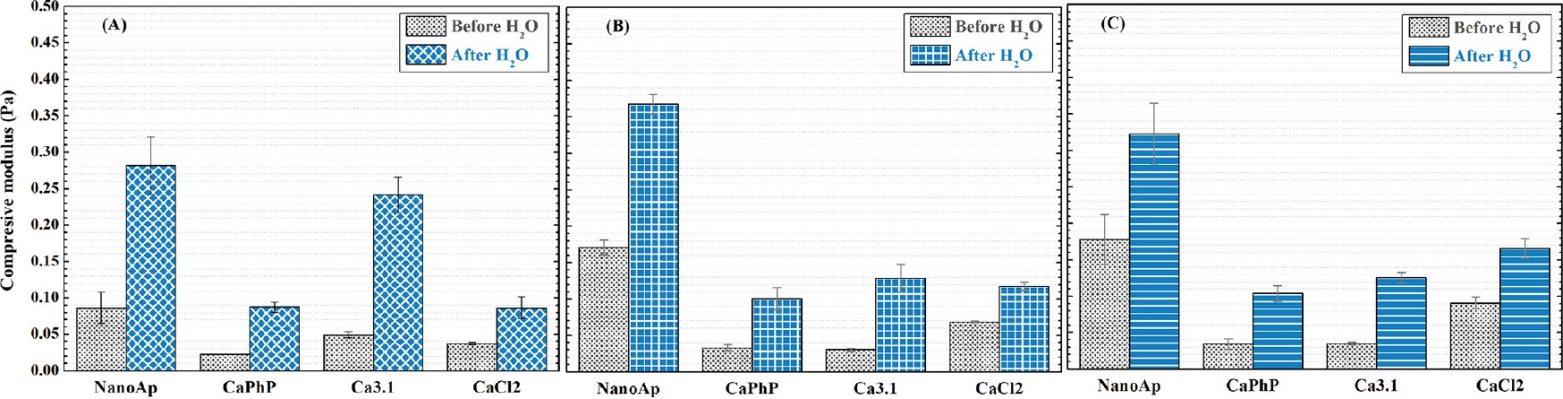
Comparison of Young’s modulus of printed samples before and after washing with respect to printing density: (A) 10% infill density, (B) 20% infill density, and (C) 30% infill density.

In order to increase the stability of the printed structures, ionic cross-linking was applied to them. The mechanical stability of hydrogels results from a variety of factors, both intrinsic (such as polymer *M*_w_ or cross-linking density) and extrinsic (shape and construction of the sample, temperature, etc.). Furthermore, fillers play a crucial role in guiding the mechanical performance. They can work as reinforcement, increasing the strength of a material, or as plasticizers. Hard inorganic particles, such as those present in the current study, generally work as reinforcing fillers due to their restricted ability to compress. Figure 11 shows the compressive moduli of NaAlg with various fillers. Nanoparticles of nanoAp failed to provide sufficient strength to the ink, and the structure collapsed during printing. Therefore, these hydrogels are excluded from the mechanical analysis.

The different infill density refers to the volume of free space in the printed cylinder. There is no distinct trend indicating correlation between infill density and compressive modulus of the hydrogels. However, there is a sharp increase of modulus (2 times or more) when the hydrogel is washed in DEMI water for several days. The values of the moduli are consistent with results published elsewhere.^48,56,57^ This treatment ensures the diffusion of excessive ions out of the hydrogel. This phenomenon is likely caused by a difference in swelling behavior with respect to the environment. The polyelectrolytic nature of NaAlg causes higher swelling in ion-rich environments. Indeed, swelling has been reported to be lower in water than in salt solutions.^58^ Also, the equilibrium swelling in CaCl_2_ solutions is dependent on their concentration, peaking at 2 wt %,^59,60^ the concentration used for the cross-linker in this study. Furthermore, the concentration of Ca^2+^ in the hydrogels is likely to induce conjugation of junction zones of α-l-guluronic acid and β-d-mannuronic acid. These junctions lead to the collapse of the polymer network, which results in a decrease of Young’s modulus.^61^ The washed samples are more suitable for mechanically demanding applications. Additionally, it is possible to assume that high salt concentration may be harmful to cells.^62^ Therefore, the highly concentrated CaCl_2_ solution is unsuitable for applications involving direct contact with living cells, specifically bioprinting, as washing is necessary from both a mechanical and a cytocompatibility point of view.

### *In Vitro* Cytotoxicity

**Figure 12:**
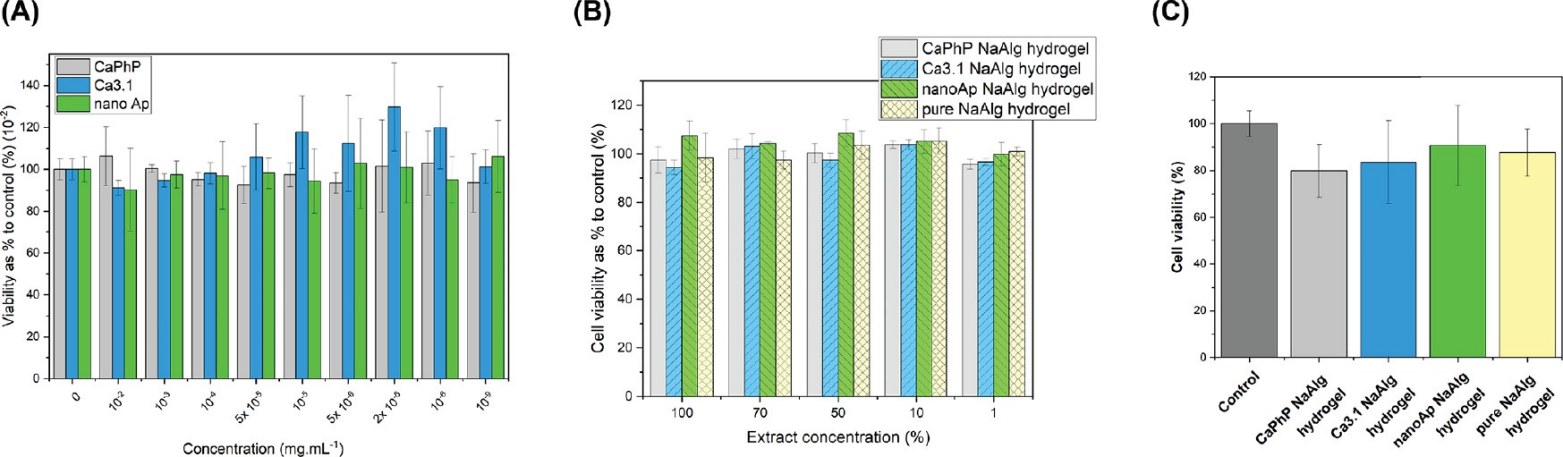
*In vitro* cytotoxicity test of (A) ethylene glycol dispersions of nanofillers after 24 h treatment of 3T3 fibroblasts, (B) extracts of NaAlg hydrogels, and (C) NaAlg hydrogels in direct contact developed in the study using 3T3 fibroblasts.

The potential application of the new hydrogels prepared in this study is as scaffolds in regenerative medicine. In this respect, the cytotoxicity of pure NPs and of fully cross-linked hydrogel formulations was assessed according to ISO norm 10993-12 using 3T3 fibroblasts and MTT assay. Three types of NPs were tested in the concentration range 10^–2^–10^–9^ mg mL^–1^. As can be seen from Figure 12A, the NPs were found to be nontoxic up to a concentration of 10^–2^ mg mL^–1^, which is 10000 times higher than the concentration necessary for rheological modification. It can be concluded that the use of all three types of NPs is safe from the cytocompatibility point of view.

Further, the cytotoxicity of different hydrogel formulations was tested after direct contact with hydrogels and as hydrogel extracts (Figure 12B,C). All prepared hydrogels were noncytotoxic. Thus, the presented materials have potential in biological applications, including scaffold fabrication and cell cultivation.

### Bioprinting

The simple usage of the bioink was possible due to the highly shear-thinning character of the pre-cross-linked inks, which imposes minimum stress on the cells during rapid mechanical mixing. The Ca3.1 nanofiller was chosen as the most suitable option due to its higher damping factor compared to CaPhP-formed pre-cross-linked ink, which is presumed to be beneficial to protecting the encapsulated cells during microextrusion 3D printing.^63^ In order to map the effect of printhead shape and size on cells, the experimental 3D bioprinting was conducted with two types of printhead tips—cylindrical needle and conical nozzle—of three different sizes: 0.52, 0.41, and 0.21 mm. It is expected that a narrower printhead causes higher shear stress and will result in lower cell viability.^64^ Additionally, the cylindrical needle imposes constant high shear stress on the material, including the cells, whereas the shear stress increases gradually with the conical nozzle.^65^ In fact, printing pressure increases with the decrease of tip diameter, as well as between nozzle and needle, which is in agreement with theory (see Table 5).

**Table 5:** Bioprinting Conditions

printhead tip	cylindrical needle			conical nozzle		
diameter (mm)	0.52	0.41	0.21	0.52	0.41	0.21
printing pressure (kPa)	46.9	51.9	82.6	88.7	119.7	178.0
stabilization with Fe^3+^	stable	stable	unstable	stable	stable	unstable

Stabilization of the NaAlg pre-cross-linked inks with 2 wt % CaCl_2_ solution is incompatible with living cells, as the high salt concentration is cytotoxic.^62^ Therefore, an alternative final cross-linking method was proposed: 0.1% MCl_*x*_ solution in PBS, where M stands for a metal cation: Zn^2+^, Mg^2+^, or Fe^3+^. The results can be found in the Supporting Information (Figure S5). Because of good long-term stability, FeCl_3_ was chosen for the stabilization of the bioprinted structures. The significant decrease of salt concentration would allow the washing out step described earlier to be omitted, enabling (a) the achievement of maximum mechanical strength in one step and (b) the immediate cultivation of cells placed on the surface of the printed structures or encapsulated in the printing material itself. In other words, the alteration of the full cross-linking procedure is a step toward preparation of a bioink. Additionally, Fe^3+^ appears more advantageous than Ca^2+^ on account of its improved mechanical stability, stimuli-responsiveness, and redox properties.^66^ However, the need to significantly reduce the concentration of multivalent ions in the cross-linking solution leads to a lower rate of final cross-linking and a 4-fold increase of cross-linking time from 30 min to 2 h. This prolonged exposure to a high-water-content environment caused dissolution of the pre-cross-linked inks in some cases (see Table 5).

It was observed that printed strands of lower dimensions disintegrate faster than bulky structures, and the prints obtained by a 0.21 mm diameter tip all dissolved before live/dead assay could be performed. Previously, it was found that porous structures formed of Ca3.1-modified materials tend to collapse during the final cross-linking (see Table 4). This led to the creation of bulkier printed structures, decreasing the disintegration rate.

**Figure 13:**
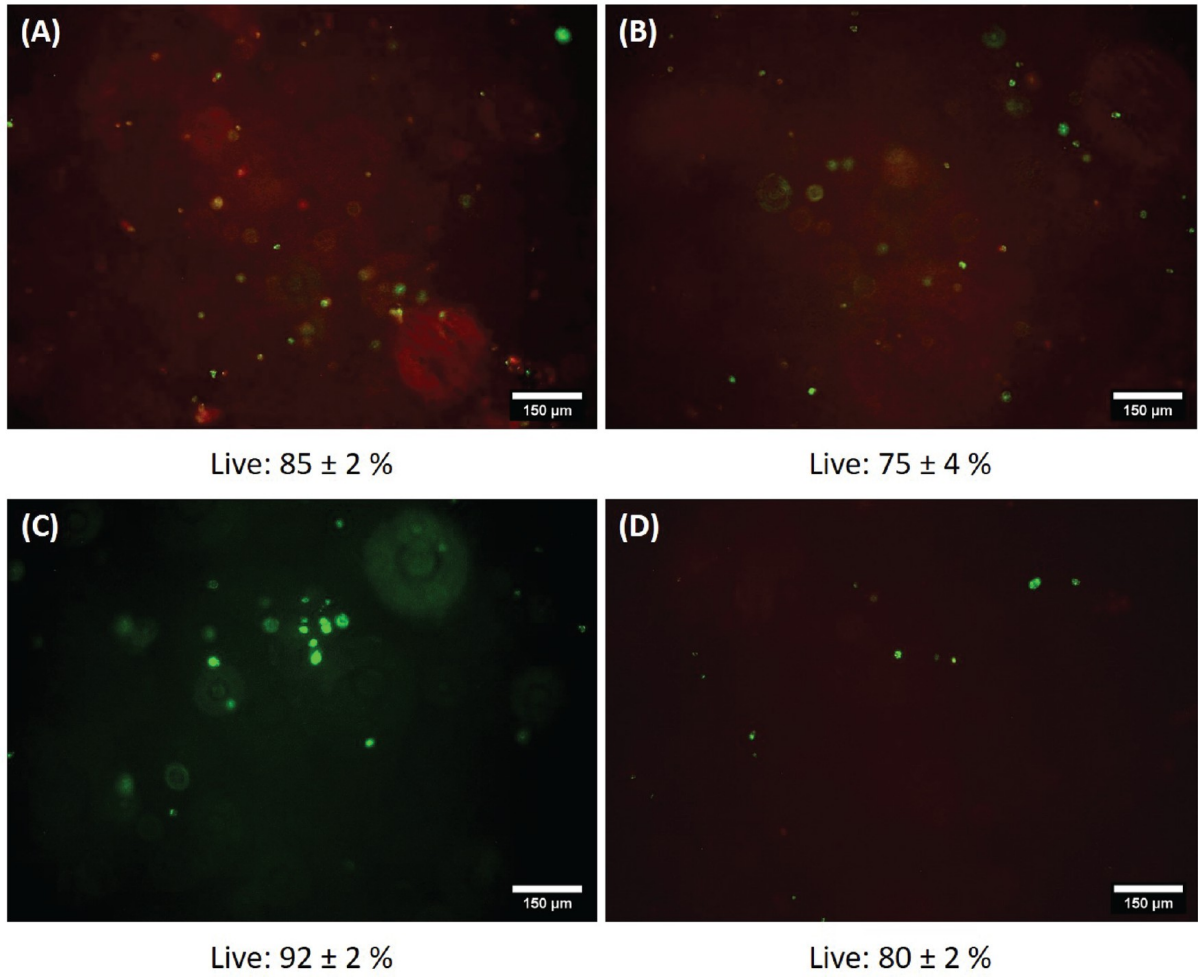
Live/dead staining of bioinks after 3D printing: (A) 0.52 mm diameter nozzle, (B) 0.52 mm diameter needle, (C) 0.42 mm diameter nozzle, and (D) 0.42 mm diameter needle.

Live/dead staining was used as a qualitative assessment of the materials’ usability as bioink. Ethidium homodimer, a DNA stain, cannot penetrate an intact cell membrane;^68^ thus, it can be used as an efficient means to detect mechanical damage resulting from microextrusion.^69^ It can be seen that extrusion printing with BALB/3T3 cells sustains 70–90% cell viability (Figure 13). Therefore, BALB cells can be safely encapsulated in Ca3.1-modified NaAlg, forming a bioink for printing via microextrusion.

## Conclusion

The current study aimed to employ layered nanoparticulate organophosphonate-based fillers in order to achieve rheological modification of NaAlg solution through the formation of a house-of-cards structure. In order to increase the hydrophilicity of NPs, enhancing the interactions in aqueous NaAlg solution, a novel material, Ca3.1, was synthesized. Its ability to modify the flow behavior of the polymer solution was compared to that of previously developed layered CaPhP NPs as well as to spherical nanoAp. Furthermore, the effect of Ca^2+^ dissociation was assessed by omitting the nanofiller carrier and using free Ca^2+^ ions in solution.

It was found that spherical nanoAp does not have any effect on NaAlg rheology as it is incapable of spontaneously creating the desired random arrangement. Free Ca^2+^ ions, however, lead to significant stiffening of the polymer, which causes overgelation and loss of printing precision. Therefore, the use of layered nanofillers—CaPhP and Ca3.1—shows potential to create highly precise constructs by microextrusion 3D printing.

In order to maintain the long term stability of the hydrogels, a secondary cross-linking using multivalent ions (Ca^2+^ or Fe^3+^, respective of a single-step or multistep cell cultivation procedure) was used. This cross-linking led to a certain shrinkage of the hydrogel and the loss of a portion of printing-induced porosity. It also led to a decreasing of stiffness in compression. However, this loss of stiffness was reversible by extracting the excess Ca^2+^ ions.

In summary, we have described the synthesis of novel layered organophosphonate NPs, as well as their use as rheological modifiers for biopolymer-based hydrogels. We have shown that these layered organophosphonates provide high printing precision in microextrusion experiments. Furthermore, we have used the NaAlg-based matrices modified with Ca3.1 for the encapsulation of BALB/3T3 cells and experimental microextrusion bioprinting. This process was proved to be nonharmful. Thus, the potential to use the materials described in this study for bioprinting is proved.
