# Supplementary material for: Synthesis and Exfoliation of Calcium Organophosphonates for Tailoring Rheological Properties of Sodium Alginate Solutions: A Path toward Polysaccharide-Based Bioink
Source: Biomacromolecules. 2023 May 30;24(7):3016–31. doi: 10.1021/acs.biomac.3c00081 (PMC10336848; doi:10.1021/acs.biomac.3c00081)
Supplement: Supplementary file 1 — bm3c00081_si_001.pdf [file bm3c00081_si_001.pdf]

Supporting Information

Synthesis and exfoliation of calcium phosphonates for tailoring rheological properties of sodium alginate solutions: A path towards polysaccharide based bioink

Kateřina Kopecká,[†,‡,¶] Lenka Vítková,[*,§] Zuzana Kroneková,[‖,⊥] Lenka Musilová,[§,#] Petr Smolka,[§,#] Filip Mikulka,[§,#] Klára Melánová,[¶] Petr Knotek,[‡] Martin Humeník,[@] Antonín Minařík,[§,#] and Aleš Mráček[*,§,#]

†SYNPO, a.s., S. K. Neumanna 1316, 532 07 Pardubice, Czech Republic

‡Department of General and Inorganic Chemistry, Faculty of Chemical Technology, University of Pardubice, Studentská 573, 53210 Pardubice, Czech Republic

¶Joint Laboratory of Solid State Chemistry, Faculty of Chemical Technology, University of Pardubice, Studentská 84, 53210 Pardubice, Czech Republic

§Department of Physics and Materials Engineering, Faculty of Technology, Tomas Bata University in Zlin, Vavrečkova 5669, 76001 Zlín, Czech Republic

‖Polymer Institute of Slovak academy of Sciences, Dúbravská cesta 9, 84541 Bratislava, Slovak Republic

⊥National Institute of Rheumatic Diseases, Nábrežie I. Krasku 4, 921 12 Piešťany, Slovak Republic

#Centre of Polymer Systems, Tomas Bata University in Zlin, tř. Tomáše Bati 5678, 76001 Zlín, Czech Republic

@Lehrstuhl Biomaterialien, Universität Bayreuth, Prof.-Rüdiger-Bormann Straße 1, 95447 Bayreuth, Germany

E-mail: vitkova@utb.cz; mracek@utb.cz

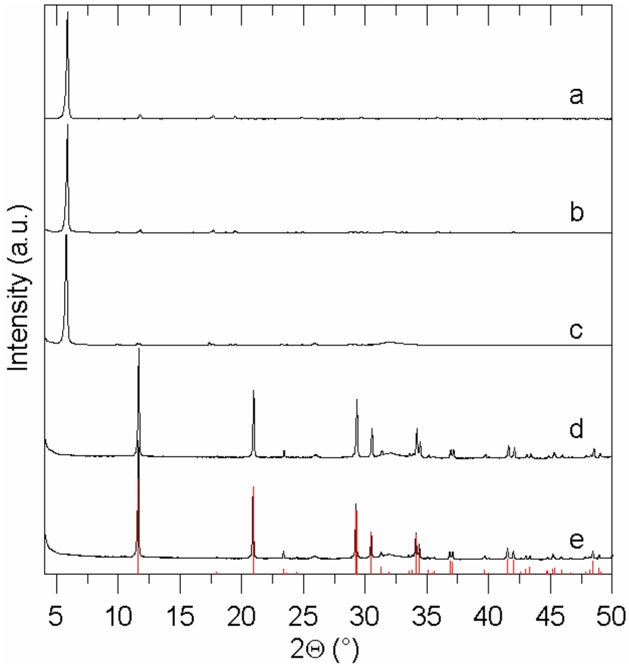

Figure S1. Powder X-ray patterns of phosphonates prepared a) CaPhP b) Ca3.1 c) Ca1.1 d) Ca1.3 e) Ca0.1. The diffraction lines of brushite (JCPDS No. 04-013-3314)[1] are marked in red.

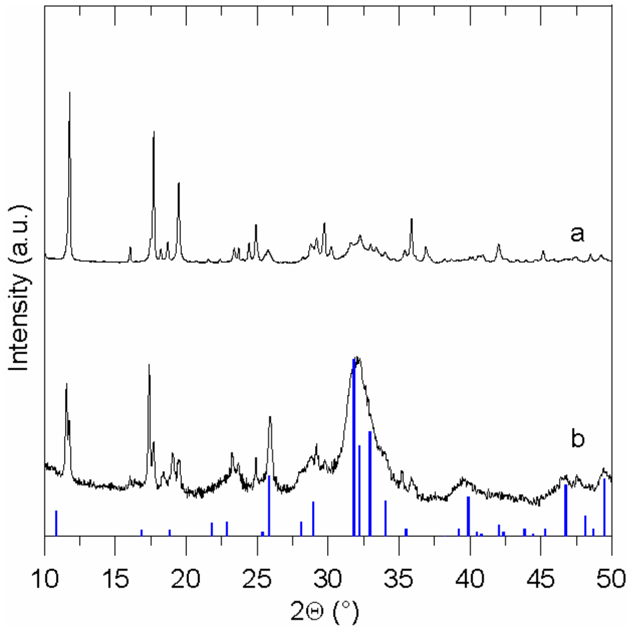

Figure S2. Parts of powder X-ray patterns a) Ca3.1 and b) Ca1.1 prepared in an ice bath. The diffraction lines of hydroxylapatite (JCPDS No. 04-007-2837)[1] are marked in blue.

Table S1. Powder pattern of **Ca3.1** indexed in monoclinic cell:

Space group: $P2_1/c$

a = 19.3258 ± 0.0003 Å

b = 11.0287 ± 0.0003 Å

c = 5.7158 ± 0.0001 Å

β = 129.055 ± 0.004°

V = 946.034 ± 0.089 $Å^3$

2Θ	d [Å]	Intensity [%]	h	k	l	$2\Theta_{exp}-2\Theta_{calc}$
5.881	15.01690	100	1	0	0	-0.004
9.942	8.88975	1	1	1	0	-0.003
11.784	7.50360	3	2	0	0	0.000
16.058	5.51511	1	0	2	0	-0.002
17.486	5.06764	1	-2	1	1	0.008
17.717	5.00206	2	3	0	0	0.001
18.218	4.86566	1	-3	1	1	0.011
18.699	4.74169	1	-1	1	1	-0.005
19.470	4.55553	2	3	1	0	0.001
21.575	4.11551	1	0	1	1	0.011
22.399	3.96602	1	-2	2	1	0.001
23.380	3.80176	1	-1	2	1	0.004
23.701	3.75097	1	4	0	0	0.006
24.423	3.64175	1	-5	1	1	0.001
24.914	3.57111	1	1	3	0	-0.003
25.574	3.48039	1	1	1	1	0.034
25.765	3.45496	1	0	2	1	0.020
28.210	3.16080	1	-5	2	1	0.005
28.785	3.09898	1	4	2	0	0.028
29.191	3.05684	1	1	2	1	0.002
29.742	3.00141	1	5	0	0	0.001
30.214	2.95560	1	2	1	1	-0.014
31.591	2.82988	1	-5	0	2	0.001
			0	3	1	0.016
32.242	2.77423	1	-6	2	1	-0.007
33.002	2.71199	1	-7	0	1	0.008
			-6	0	2	0.012
			1	4	0	-0.003
33.385	2.68176	1	2	2	1	-0.024
34.013	2.63371	1	-7	1	1	0.005
			-6	1	2	0.009
			5	2	0	0.034
35.403	2.53339	1	-7	0	2	0.007
			-4	2	2	0.022
			3	1	1	0.014
35.871	2.50143	1	6	0	0	-0.003
36.899	2.43404	1	-7	2	1	0.000
			-6	2	2	0.004
40.558	2.22247	1	-1	2	2	-0.007
			4	4	0	-0.014
40.888	2.20532	1	0	5	0	0.008
			1	4	1	-0.005
			4	1	1	-0.008
42.011	2.14892	1	-8	2	1	0.008
42.529	2.12393	1	3	3	1	0.021
43.935	2.05919	1	-2	5	1	-0.038
			0	2	2	-0.008
44.601	2.02997	1	5	4	0	0.012

45.147	2.00667	1	-9	1	1	-0.003
45.903	1.97536	1	-5	4	2	-0.012
			0	5	1	-0.002
			5	0	1	-0.007
47.452	1.91443	1	-9	2	1	-0.017
			-5	5	1	0.013
48.484	1.87608	1	-6	1	3	-0.023
			-5	0	3	0.005
			8	0	0	-0.004
49.231	1.84935	1	-5	1	3	0.009
			8	1	0	0.000
51.140	1.78470	1	-10	1	1	0.012
			-9	1	3	0.003
			-9	3	1	-0.016
			2	6	0	0.019
52.235	1.74985	1	-3	0	3	0.005
			-2	6	1	-0.006

Indexation was done using DICVOL91 program from the CRYSFIRE software package.[2] The space groups were determined by processing the data in the Checkcell program.[3]

a) b)

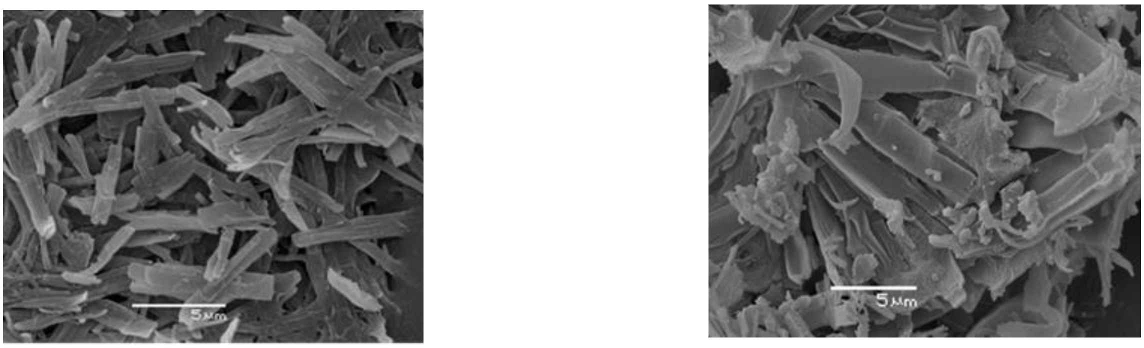

Figure S3. SEM images of a) CaPhP and b) Ca3.1.

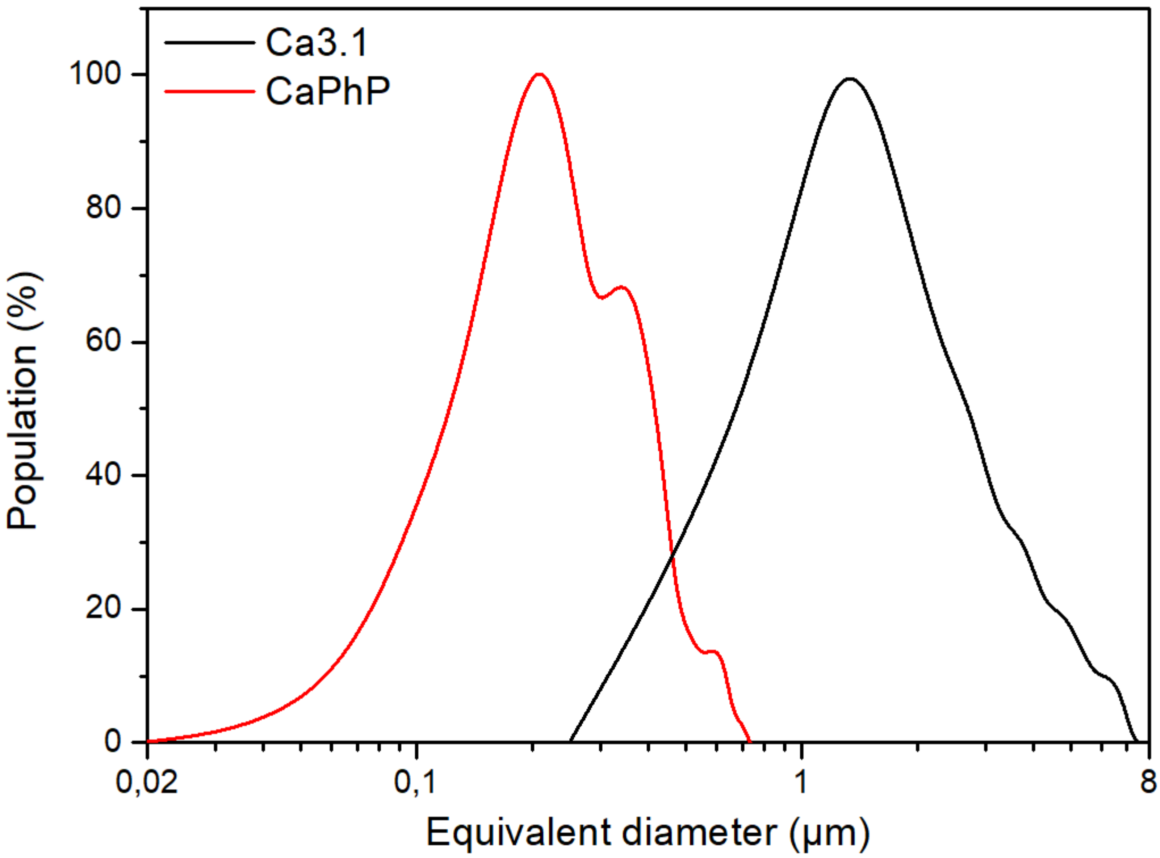

Figure S4. The histograms of the primary particle size distribution based on the AFM images.

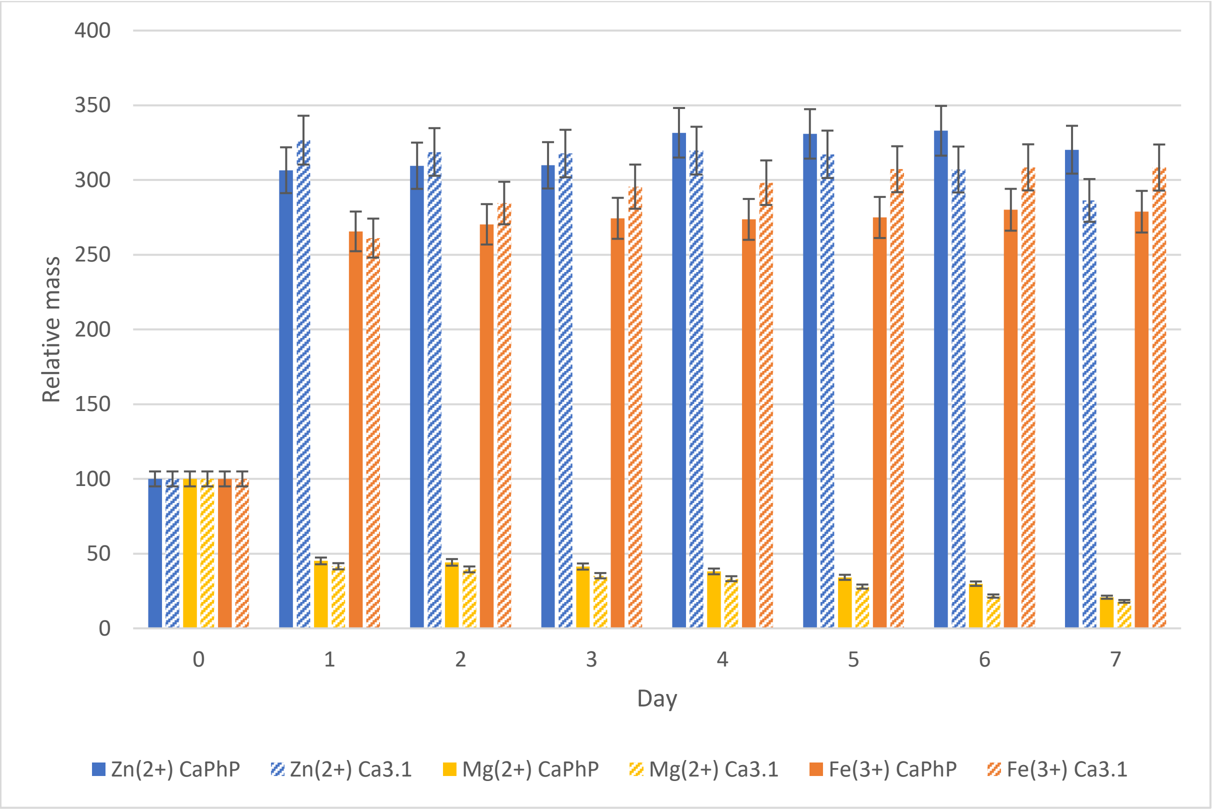

Figure S5. The stability of NaAlg hydrogels modified with CaPhP or Ca3.1 particles and crosslinked by Zn^{2+}, Mg^{2+} or Fe^{3+} kations. The stability is determined as the relative mass of the sample over the course of 7 days.

References

1. International Centre of Diffraction Data, *Joint Committee on Powder Diffraction Standards*, Swarthmore, PA., USA.
2. R. Shirley, (2002), "The crysfire 2002 system for automatic powder indexing: user's manual", The Lattice Press, 41 Guildford Park Avenue, Guildford, Surrey GU2 7NL, England.
3. J. Laugier and B. Bochu, LMGP-Suite; ENSP/Laboratoire des Materiaux et du Genie Physique, BP 46. 38042 Saint Martin d'Heres, France.
